# Unveiling the Important Role of Gut Microbiota and Diet in Multiple Sclerosis

**DOI:** 10.3390/brainsci15030253

**Published:** 2025-02-27

**Authors:** Amina Džidić Krivić, Emir Begagić, Semir Hadžić, Amir Bećirović, Emir Bećirović, Harisa Hibić, Lejla Tandir Lihić, Samra Kadić Vukas, Hakija Bečulić, Tarik Kasapović, Mirza Pojskić

**Affiliations:** 1 Department of Neurology, Cantonal Hospital Zenica, Crkvice 67, 72000 Zenica, Bosnia and Herzegovina; amina.dzidickrivic@gmail.com (A.D.K.); tandirlejla30@gmail.com (L.T.L.);; 2 Department of Physiology, School of Medicine, University of Zenica, Travnička 1, 72000 Zenica, Bosnia and Herzegovina; 3 Department of Neurosurgery, Cantonal Hospital Zenica, Crkvice 67, 72000 Zenica, Bosnia and Herzegovina; begagicem@gmail.com (E.B.);; 4 Department of Doctoral Studies, School of Medicine, University of Tuzla, 75000 Tuzla, Bosnia and Herzegovina; 5 Internal Medicine Clinic, University Clinical Center of Tuzla, Ulica prof. dr. Ibre Pašića, 75000 Tuzla, Bosnia and Herzegovinabecirovic.emir@live.com (E.B.);; 6 Department of Physiology, School of Medicine, University of Tuzla, Univerzitetska 1, 75000 Tuzla, Bosnia and Herzegovina; 7 Department of Maxillofacial Surgery, Cantonal Hospital Zenica, Crkvice 67, 72000 Zenica, Bosnia and Herzegovina; 8 Department of Neurology, School of Medicine, University of Zenica, Travnička 1, 72000 Zenica, Bosnia and Herzegovina; 9 Department of Anatomy, School of Medicine, University of Zenica, Travnička 1, 72000 Zenica, Bosnia and Herzegovina; 10 Department of Neurosurgery, University Hospital Marburg, Baldingerstr., 35033 Marburg, Germany

**Keywords:** multiple sclerosis, gut microbiota, gut dysbiosis, diet, probiotics

## Abstract

Multiple sclerosis (MS) is a chronic inflammatory disease of the central nervous system (CNS), characterized by neurodegeneration, axonal damage, demyelination, and inflammation. Recently, gut dysbiosis has been linked to MS and other autoimmune conditions. Namely, gut microbiota has a vital role in regulating immune function by influencing immune cell development, cytokine production, and intestinal barrier integrity. While balanced microbiota fosters immune tolerance, dysbiosis disrupts immune regulation, damages intestinal permeability, and heightens the risk of autoimmune diseases. The critical factor in shaping the gut microbiota and modulating immune response is diet. Research shows that high-fat diets rich in saturated fats are associated with disease progression. Conversely, diets rich in fruits, yogurt, and legumes may lower the risk of MS onset and progression. Specific dietary interventions, such as the Mediterranean diet (MD) and ketogenic diet, have shown potential to reduce inflammation, support neuroprotection, and promote CNS repair. Probiotics, by restoring microbial balance, may also help mitigate immune dysfunction noted in MS. Personalized dietary strategies targeting the gut microbiota hold promise for managing MS by modulating immune responses and slowing disease progression. Optimizing nutrient intake and adopting anti-inflammatory diets could improve disease control and quality of life. Understanding gut-immune interactions is essential for developing tailored nutritional therapies for MS patients.

## 1. Introduction

**Figure 1:**
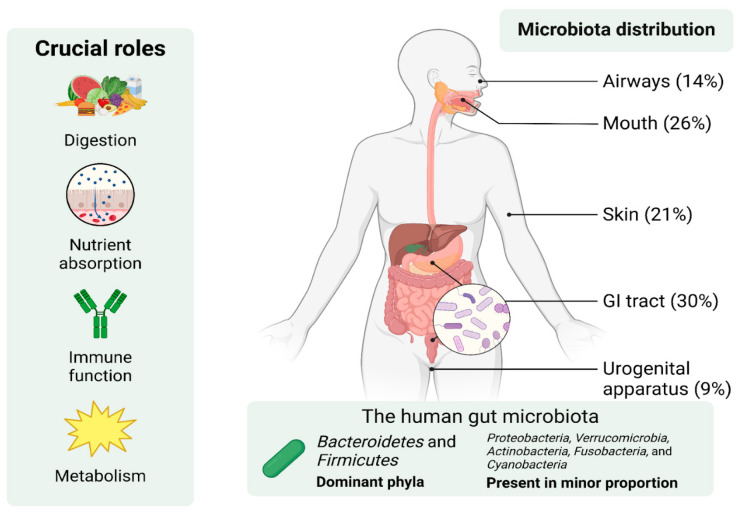
The essential roles and anatomical distribution of human microbiota, with their involvement in digestion, nutrient absorption, immune function, and metabolism. The distribution of microbiota across body regions shows the highest concentration in the gastrointestinal (GI) tract (30%), followed by the mouth (26%), skin (21%), airways (14%), and urogenital apparatus (9%). The human gut microbiota is dominated by *Firmicutes* and *Bacteroidetes*, with smaller proportions of other bacterial phyla such as *Proteobacteria*, *Verrucomicrobia*, *Actinobacteria*, *Fusobacteria*, and *Cyanobacteria*. GI, gastrointestinal tract. Adopted from [7]. Created in BioRender. Hadzic, S. (2025) https://BioRender.com/a99u000 (accessed on 26 December 2024).

Gut microbiota is an assortment of microorganisms inhabiting the mammalian intestines. They have a crucial role in different aspects of the host’s health, including digestion, nutrient absorption, the functioning of the immune system, and metabolism [1,2]. Microbial communities inhabit every part of the human body, as shown in Figure 1, with particularly dense colonization occurring in the intestines, forming what is known as the microbiota or commensal microflora [3,4,5]. The host and its gut microbiota together form a complex entity known as the ‘superorganism’, combining human and bacterial genes [6,7,8]. Over 1000 distinct bacterial species and approximately 1014 bacterial cells are thought to be present in the adult human gut [8]. Furthermore, recent studies have noted that gut microbiota can influence both distant and nearby organ systems, thus making it an important factor in the onset or progression of a variety of disorders [2,9]. Maintaining the host’s homeostasis depends on the general balance of the gut microbial community’s composition, as well as the existence or lack of significant species that influence the host’s health [1,10].

Aerobes and facultative anaerobes are two to three orders of magnitude inferior to stringent anaerobes, which make up the bulk of the gut microbiota, according to [1]. The number of bacterial species found in the human gut has been estimated to be between 500 and 1000, although estimates vary greatly throughout studies [1,6]. There are also more than 50 bacterial phyla that have been described to date, including *Proteobacteria*, *Verrucomicrobia*, *Actinobacteria*, *Fusobacteria*, and *Cyanobacteria*, that are present in trace amounts. Hence, the human gut microbiota is dominated by two of them: the *Bacteroidetes* and the *Firmicutes* [1,2]. Collectively, all microbes and their genetic components are marked as the ’microbiome’. Estimates suggest that the microbiome contains 100 times more genes than the human genome [2]. They serve as a crucial barrier against colonization by harmful microbes, a concept known as colonization resistance [2,3]. Recent studies also underscore the gut microbiota’s important role in immune function and host energy metabolism [2,11]. These include maintaining the integrity of the mucosal immune system and intestinal epithelial balance [4,6].

### Main Factors Altering the Composition of Gut Microbiota

**Figure 2:**
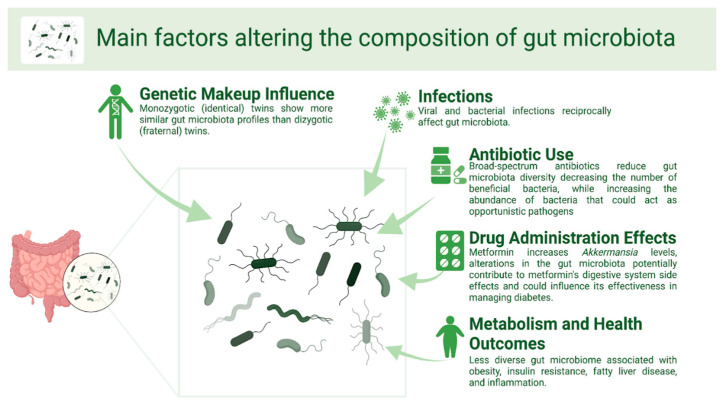
Gut microbiota can be influenced by numerous intrinsic and extrinsic factors. Created in BioRender. Hadzic, S. (2025) https://BioRender.com/f02l310 (accessed on 26 December 2024).

A wide palette of internal and external variables affects the composition of the gut microbiota, as shown in Figure 2. Among them is the host’s genetic composition [12]. Family members exhibit greater similarity in microbiota communities compared to unrelated individuals, with monozygotic twins showing more similar gut microbiota profiles than dizygotic twins [12,13,14]. Infections, both viral and bacterial, also have a reciprocal relationship with the gut microbiota [15,16,17]. Chou et al. [15] registered that the clearance of hepatitis B virus infection in a mouse model depends on the renewal of the healthy gut microbiota. The alteration of gut microbiota was also noted in studies investigating the effects of an enteropathogenic infection with *Citrobacter rodentium* on the microbiota composition in mice [17], as well as in a study investigating the *Clostridium difficile* infection that noted reduced microbial diversity in infected patients when compared to the healthy subjects [18]. Also, *C. difficile* infection is a typical result of severe dysbiosis in the gut microbiota [19,20,21,22].

The gut microbiota is also shaped by different types of drugs, especially antibiotics [23,24]. Broad-spectrum antibiotics decrease bacterial diversity and the number of beneficial bacteria, while increasing the abundance of potentially opportunistic bacteria [25]. For example, the administration of clindamycin has been linked with the longest-lasting effects on the gut microbiota [26,27]. Gut dysbiosis caused by early antibiotic exposure in neonates is a potential risk factor for inflammatory bowel disease later in life [28,29,30]. Also, antibiotic usage in children could contribute to obesity later in life by altering the gut flora, according to studies conducted in both people and animals [31,32,33,34]. Other studies demonstrated that antibiotics could reduce body weight and increase insulin sensitivity by modifying gut bacteria [35,36]. For example, this significantly contributes metabolically to the host organism by fermenting complex indigestible dietary carbohydrates and proteins, creating short-chain fatty acids as fermentation by-products. Additionally, they are pivotal in synthesizing vitamins, facilitating ion absorption, and converting dietary polyphenolic compounds into their bioactive forms [10,11]. Therefore, while the role of antibiotics in the rising rates of obesity, particularly in childhood, remains unclear, their profound impact on gut microbiota composition is undeniable.

This is also confirmed by various products that have shown an antidiabetic property by changing the gut flora, such as berberine [37,38]. Also, metformin is commonly prescribed to treat Type 2 diabetes, and it has recently been noted that metformin administration can also modify the composition of the gut [39,40,41]. For instance, in a study inducing obesity in mice through a high-fat diet, metformin increased levels of *Akkermansia*, a bacterium known for degrading mucin, and more prominently in obese mice [42]. Similarly, recent human studies have confirmed metformin’s impact on the gut microbiota [41]. This indicates that alterations in the gut bacteria potentially contribute to metformin’s digestive system side effects and could influence its effectiveness in managing diabetes.

In addition, drugs that are often used for the treatment of MS relapse, marked as disease-modifying treatments (DMTs), aim to decrease relapse rates and MS progression. DMTs, including interferon-β (IFN-β), fingolimod, and ocrelizumab, increase the gut microbiota diversity and reduce pro-inflammatory bacteria. This indicates their influence on the gut microbiota composition and their potential use as a mechanism that improves their therapeutic effects. Also, as a recent systematic review noted, DMTs do not always impact the diversity of the microbiota as a whole, but they do lead to variations at the taxonomic level, thus, having consequences on the course of the disease. The study argued their potential role in modulating disease activity in MS patients through microbiome alterations, which should be confirmed in further studies and larger clinical trials [43].

The role of food-ingested bacteria in shaping the gut microbiome was previously underestimated, largely due to methodological limitations that have since been addressed [43]. Extensive research in both mice and humans has established that high-calorie diets contribute significantly to conditions like obesity and Type 2 diabetes [44,45]. However, emerging evidence indicates that the critical connection between diet and these conditions lies within the gut microbiota [46,47]. Human gut microbiota has been described by 16S ribosomal RNA sequencing studies as various enterotypes based on the types of bacteria present. These enterotypes have been significantly linked to long-term diets, especially diets high in protein and animal fat [48,49]. In their study, Wu et al. [50] demonstrated that diets rich in protein and animal fat correlated with higher levels of *Bacteroides*, whereas carbohydrate-rich diets were linked with *Prevotella*. Additionally, they conducted controlled feeding experiments with 10 subjects, observing that the gut microbiome changed after 24 h of initiating either a high-fat, low-fiber diet or a low-fat, high-fiber diet [50]. These findings strongly suggest that diet has a key role in shaping enterotype distribution within the gut microbiota.

Therefore, recognizing diet as a pivotal factor in gut microbiome creates an opportunity for intervention. Interventional studies have demonstrated that dietary modifications cause rapid and substantial alterations in gut microbiome [48]. The Western diet has been implicated as a significant contributor to this phenomenon. Also, yogurt consumption is perceived as beneficial to health, potentially due to its ability to modify the host gut microbiota alongside other nutritional factors [51]. Despite limited studies for decades, recent advancements in high-throughput sequencing technology and bioinformatics have led to a surge in research, including clinical trials over the past eight years, investigating how yogurt consumption influences changes in the gut microbiota [52,53]. Recent studies suggest that consuming probiotic bacteria in yogurt and other fermented dairy products positively impacts the host’s gut microbiome [12]. Fermented dairy products introduce numerous lactic acid bacteria into the gastrointestinal tract, potentially altering the intestinal environment by reducing lipopolysaccharide production and enhancing gut epithelial cell integrity through increased tight junction formation [12]. Another potential avenue of gut microbiota altering is the transplantation of the gut microbiome from healthy donors with the aim of promoting the abundance of healthy bacteria [19,20]. However, it still remains to be determined whether sustained dietary modifications can establish enduring changes in bacterial enterotypes and even be used as a potential novel therapeutic approach to the treatment of gut dysbiosis. Developing specialized dietary therapies for MS patients requires an understanding of the gut–brain axis. Namely, through immune response modulation, personalized dietary interventions targeting the gut microbiota could be beneficial for patients with MS. Adopting anti-inflammatory diets and optimizing nutritional habits has the potential to enhance quality of life (QoL) for MS patients, as well as to help with disease management. Further studies and larger clinical trials are required to provide a more thorough understanding of complex interactions between gut microbiota and MS onset and progression. The clinical implications of these findings hold the promise of effective dietary treatments and microbiota-targeted drugs as supplemental techniques in the management of MS.

## 2. The Complex Relationship Between Gut Microbiota and Multiple Sclerosis

Recently, MS has been associated with gut microbiota dysbiosis [54,55]. It is a complex neurological disease defined by chronic inflammation and immune-mediated damage to the central nervous system (CNS). Increasing incidence of MS cases in the last 10 years is most likely influenced by environmental factors, including changes in dietary habits that impact the gut microbiome [56,57,58,59,60]. Research into the association between gut microbiota, diet, and the immune system in MS has recently garnered significant interest. This complex relationship underscores the challenge of investigating specific dietary components that influence the gut–brain axis.

### 2.1. Gut Microbiota Composition in MS Patients

Initial proof of gut dysbiosis in MS was observed in a Japanese population, coinciding with a recent rise in MS cases in Japan [61]. It is proposed that the main cause of gut dysbiosis is dietary change [62]. Namely, Westernized lifestyles and diets, known for promoting inflammation in the gut [57], have been implicated as a key factor in increasing MS risk. Conversely, adopting a healthy diet has shown potential to promote intestinal microbiota towards an anti-inflammatory status, supporting dietary interventions in MS management [57,63]. Additionally, studies have repeatedly noted that patients with MS have a distinct composition of gut microbiota compared to healthy patients [60,61]. These findings indicate that particular microbial communities can trigger harmful immune responses in individuals with MS. Modifying the gut microbiota to restore balance and improve immune control shows potential as a treatment for MS, such as through probiotics, prebiotics, and dietary treatments [58,59,60]. However, further larger clinical trials should confirm these findings.

### 2.2. Underlying Mechanisms of Gut Microbiota Interactions with the Immune System in MS

**Figure 3:**
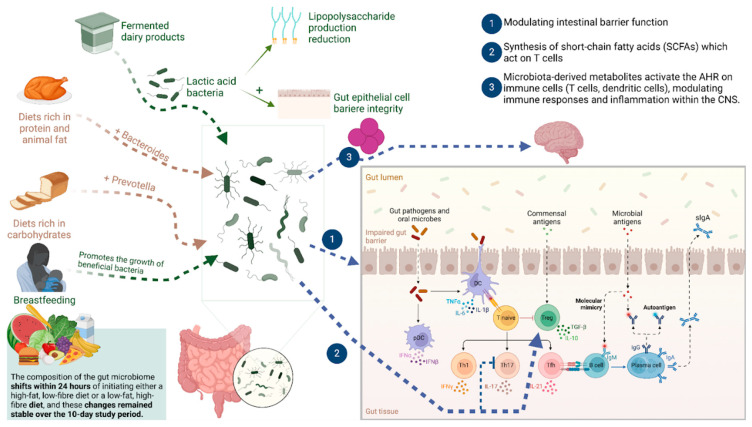
Gut microbiota is a dynamic ecosystem that responds to various intrinsic and extrinsic factors, changing the composition of bacteria inside the gut, which is shown in the left part of Figure 3. Present bacteria then influence intestinal barrier function or produce molecules that act on the cells of the immune system modulating its activity, as represented in the right part of the figure. SCFAs, short-chain fatty acids; CNS, central nervous system; AHR, Aryl hydrocarbon receptor; LPS, Lipopolysaccharides; T cells, T lymphocytes; LAB, lactic acid bacteria. Adopted from [6]. Created in BioRender. Hadzic, S. (2025) https://BioRender.com/.

One of the most significant mechanisms underlying the interplay between gut microbiota and the immune system is via modulating the intestinal barrier function [64,65,66]. The gut barrier is composed of tightly organized epithelial cells, responsible for preventing pathogens and toxins from entering the bloodstream [67,68]. Gut dysbiosis can disrupt this tight barrier, leading to a condition marked as “leaky gut”. This enables microbial antigens and other pro-inflammatory molecules to enter the circulation system, triggering immune responses that can contribute to systemic inflammation and the onset of autoimmunity [69]. Berer et al. [70] noted that the gut microbiota derived from patients with MS also triggers the development of spontaneous autoimmune encephalomyelitis in mice [70], indicating that gut dysbiosis promotes inflammatory immune response, as shown in Figure 3. There are fewer favorable bacteria, such as *Bacteroides*, as well as more potentially harmful bacteria, like *Akkermansia* and *Methanobrevibacter*, consequently affecting the function of immune cells, like regulatory T cells (Tregs) and Th17 cells, in fulfilling their role of keeping the immune system in check and stopping autoimmunity onset [62]. For example, Cekanaviciute et al. [71] discovered that the gut bacteria of MS patients can influence the functions of T cells and exacerbate the MS symptoms in animal models [71]. Furthermore, the gut bacteria also regulates the formation of short-chain fatty acids (SCFAs) by degrading dietary fibers to create SCFAs, such as propionate, butyrate, and acetate, which have immunomodulatory properties [72].

In addition, the reciprocal relationship between the intestines and the CNS, marked as the gut–brain axis, highlights the influence of gut bacteria on MS. This axis includes neurological, hormonal, and immunological processes that enable the intestines to affect CNS function and vice versa [73]. For example, Thirion et al. [74] discovered notable changes in gut microbiota composition in MS patients. However, the main question that remains to be further researched is focused on investigating whether the gut dysbiosis is a cause or a consequence of MS. Hence, dysbiosis is linked to MS through various alterations of the human immune system, with alterations of gut microbiota having a key role in promoting inflammation. Consequently, this could trigger the onset or exacerbation of MS. On the other hand, MS itself can promote gut dysbiosis through changes in the gut–brain axis, as well as due to the use of various immunomodulatory drugs in the MS treatment. This could indicate that dysbiosis is a secondary effect of MS. Nevertheless, the current opinion is that the link between gut dysbiosis and MS is bidirectional [74,75,76]. Therefore, their relationship is complex, and a better understanding could lead to the engineering of novel treatments for MS, mostly focused on inflammation reduction.

## 3. Therapeutic Potential of Gut Microbiota Modulation in MS

Gaining a comprehensive understanding of gut microbiota and its relationship with immunity is essential for the development of precise and focused therapies for both adult and pediatric patients with MS [76,77,78,79,80,81,82]. An important mechanism includes the synthesis of SCFAs that have demonstrated anti-inflammatory properties via interacting with G protein-coupled receptors (GPCRs), like GPR41 and GPR43 on immune cells [72]. Research indicates that SCFAs can enhance the development of regulatory T cells (Tregs), responsible for preserving immunological tolerance and inhibiting autoimmune reactions [62,72]. Also, aryl hydrocarbon receptor (AHR), a ligand-activated transcription factor expressed on different immune cells, including T cells and dendritic cells, interacts with gut microbiota, thus having the potential to modulate immune responses and inflammation within the CNS [83,84,85]. Moreover, activation of the AHR can affect the functioning of microglia in the CNS, which in turn has an impact on neuroinflammation [86].

Multiple studies have examined the capacity of probiotics to improve symptoms and slow down the progression of MS, such as the use of particular probiotic strains, including *Lactobacillus* spp. and *Bifidobacterium* spp. [87,88,89]. Prebiotics can also improve intestinal barrier integrity, reduce systemic inflammation, and modify immunological responses by encouraging the proliferation of commensal bacteria that produce SCFAs [90]. Also, a study that researched the effect of aerobic exercise with probiotic intake on the myelination of nerve fibers in a cuprizone-induced mouse model of MS noted that lifestyle interventions have the potential to alleviate inflammatory processes in the brains of MS patients [91]. Therefore, dietary interventions aimed at modifying gut microbiota composition represent a non-invasive and potentially cost-effective additional therapeutic approach to managing MS.

### 3.1. The Mediterranean Diet

The MD, initially investigated by Keys in the 1960s, mirrors the dietary habits of Mediterranean populations, emphasizing seasonal, communal eating, rest, physical activity, and local foods [92,93]. It limits consumption of dairy and meat and increases consumption of fruits, vegetables, nuts, legumes, whole grains, and fish. It also recommends the use of olive oil and moderate amounts of alcohol, particularly red wine [94]. In the general population, the MD demonstrates favorable health effects, such as a lower incidence of a number of chronic illnesses, including several neurodegenerative conditions like depression and cognitive decline [95]. This could be attributed to the diverse phytochemical compounds in its foods, known for their nutraceutical properties.

Because of their anti-inflammatory and antioxidant properties, they lower serum levels of inflammatory factors, including C-reactive protein and interleukin-(IL-) 6 [96]. Also, the lignans, tocopherols, phenolic acids, flavonoids, stilbenes, carotenoids, and unsaturated fatty acids, present in various foods within the MD, render them “functional foods”. Extra virgin olive oil (EVOO), a staple of the MD, is renowned for its rich content of phytochemical compounds, particularly polyphenols, which confer various health benefits. Its main lipid constituents are triglycerides, consisting predominantly of oleic acid (73.6%), followed by palmitic acid (13.7%) and linoleic acid (7.85%) [97]. EVOO contains over 230 bioactive molecules, including phenols, sterols, chlorophylls, carotenoids, alcohols, and esters, exhibiting neuroprotective properties by inhibiting pathways associated with inflammation and oxidative stress. Daily consumption of EVOO can provide significant health benefits, including protection against oxidative stress-related damage [98].

The function of MD in MS has been investigated in recent research [99,100,101,102,103]. The relationship between MS severity and MD adherence was examined in a single-center study involving 106 patients. According to the study, individuals who adhered to the MD had less severe symptoms of MS than patients who did not. Importantly, no individual component of the MD showed an independent association with MS severity, suggesting that the combined dietary pattern plays a synergistic role over individual food intake [92].

Researchers evaluated the effects of the Western diet (WD) and a high-vegetable/low-protein (HV/LP) diet, which is similar to the MD, in people with RRMS in a pilot trial. The gut composition of the HV/LP group had more butyrate-producing *Lachnospiraceae* bacteria, fewer pro-inflammatory PD-1+ and IL-17+ T cells, and more anti-inflammatory PD-L1+ monocytes. Clinically, during follow-up, the EDSS score and relapse rate significantly decreased in the HV/LP group. However, the number of participants was small, and larger studies are required to confirm these findings [57]. Additionally, a recent study on individuals with MS found a positive correlation between circulating Th17 cell levels and meat consumption. The meat consumption was negatively correlated with the relative abundance of *Bacteroides thetaiotaomicron*, which has a high capacity for polysaccharide digestion. *B. thetaiotaomicron* was inversely associated with circulating Th17 cells, while Th17 cells were positively linked to meat intake [104], as noted in Table 1.

### 3.2. Ketogenic Diet

The ketogenic diet (KD) is a dietary intervention based on high-fat, low-carbohydrate intake, inducing ketosis and modulating various metabolic pathways [118]. Widely used in clinical practice for drug-resistant epilepsy and inflammatory conditions like Febrile infection-related epilepsy syndrome [119]. Beta-hydroxybutyrate (BHB) and acetoacetate (ACA) represent the main ketone products generated by KD. These compounds have potential anti-inflammatory and neuroprotective effects, as they help reduce oxidative stress, support mitochondrial function, regulate epigenetic changes, and influence the gut microbiome composition [120]. KD’s mechanisms of action involve targeting immune activation pathways and inflammatory mediators, including adenosine, ketone bodies, PPAR-γ, NLRP3 inflammasome, and gut microbiota [121,122]. BHB, a key anti-inflammatory agent generated during the KD inhibits the activation of IL-1β [123] mediated by the NLRP3 inflammasome, potentially contributing to the diet’s anti-inflammatory properties [124]. Dysregulation of the inflammasome is implicated in autoimmune disorders, including MS and EAE, where NLRP3 mediates immune cell migration to the CNS, contributing to neuro-inflammation [125]. KD has been shown to improve inflammatory markers, disability, cognition, and disease progression in rats with EAE. Ketone bodies from KD provide an alternative source of energy for the brain and may reduce neuroinflammation by inhibiting NLRP3. They also enhance mitochondrial biogenesis and redox balance [125,126].

Effective dietary interventions for MS can modulate inflammation, protect against 452 neurodegenerations, or promote nervous system repair. The impact may result from direct metabolite actions, gut microbiota metabolites, or diet-mediated alterations in gut bacteria composition [58,59,60]. The nutrition plan includes two phases: adaptation and maintenance. The adaptation phase lasts four weeks with 20 g/day carbohydrate intake to establish ketosis. In the one-month maintenance phase, carbohydrate consumption increases by 5 g each week up to 40 g/day, maintaining low glycemic index and load for sustained ketosis and stable blood sugar levels. A similar nutritional approach was tested in a clinical trial, showing good compliance in MS patients, and the study noted that the KD is a cost-effective complementary therapeutic option for MS. The protocol follows international ketogenic guidelines, with a caloric deficit of 300–500 kcal varying with BMI. Daily water intake is set at 0.4 mL per kg of body weight [96].

Recent studies have noted several important effects of KD on MS pathology. Notably, the KD has been shown to reduce hippocampal demyelination, inhibit the activation of microglia and astrocytes, and modulate the SIRT1/PPAR-γ and SIRT1/P-Akt/mTOR pathways [126]. Furthermore, KD affects the expression of several enzymes implicated in MS’s inflammatory response. It suppresses the systemic production of arachidonate 5-lipoxygenase (ALOX5), cyclooxygenase (COX) 1, and COX2, which are essential for the synthesis of pro-inflammatory eicosanoids and linked to inflammation and demyelination in multiple sclerosis [106]. Furthermore, it was also reported that the KD group exhibited significantly reduced levels of serum neurofilament light chain (sNfL) compared to the common diet group [127]. These findings suggest that the KD could be a potential therapeutic strategy for managing MS through multiple pathways.

The gut microbiota of patients with MS may also be impacted by the KD. When compared to MS patients who did not follow the KD, a six-month KD intervention reduced six bacterial groups, such as *Bacteroides* and *Faecalibacterium prausnitzii*. The only bacteria that did not quickly decline after the KD intervention was *Akkermansia.* Bacterial concentrations started to recover after 12 weeks and reached levels comparable to healthy controls after 23–24 weeks, except for *Akkermansia*, which declined post-KD, as shown in Table 1 [107]. Despite these initial results, the long-term use of KD in MS patients requires careful evaluation due to potential risks, such as an increase in apoB-containing lipoproteins, which could elevate cardiovascular disease risk [128]. A recent meta-analysis of 11 studies including 608 subjects found that modified MD improved both fatigue and QoL, while low-fat diets only improved fatigue. Fasting, calorie-restricted, and anti-inflammatory diets had no significant impact, and ketogenic diets showed mixed results. However, all these studies require larger clinical trials to reach a general consensus about appropriate diet for MS, as well as their safety and efficacy [129].

### 3.3. Calorie Restriction

The term “dietary restriction” (DR) refers to controlled dietary regimens used in experimental models, involving reduced energy intake without causing malnutrition [130]. These regimens have shown promising protective effects against EAE, a widely studied model for MS [130]. Although the precise mechanisms by which DR affects the gut microbiota in MS remain largely unknown, recent studies on intermittent fasting (IF), a special type of DR, have shown improvements in EAE pathology and clinical outcomes [108,127]. The principal DR consists of a daily calorie restriction (CR) and IF involves periodic elimination or a significant reduction in food intake for specific intervals. Intermittent fasting (IF) comes in several forms, each offering a unique approach to calorie restriction [131,132,133]. IF has been associated with alterations in microbiota composition, enriching it with beneficial bacteria, such as *Lactobacillaceae*, *Bacteroidaceae*, and *Prevotellaceae*. Remarkably, fecal microbiome transplantation from IF-treated mice to mice on a normal dietary regimen has shown promising therapeutic potential in ameliorating EAE symptoms. These findings suggest a complex interplay between dietary restriction, gut microbiota, and MS pathogenesis, warranting further investigation into their potential therapeutic implications [111]. IF also reduced the accumulation of monocytes in the spinal cord of EAE mice. Monocytes from mice that fasted had downregulated expression of pro-inflammatory genes such as TNF-a, IL-1b, CXCL2, and CXCL10 compared to those from non-fasted mice [115].

In a study by Fitzgerald et al. [134], the safety and practicality of various CR diets were researched in 36 patients with MS. It was noted that traditional CR led to greater weight loss compared to IF, although adherence was lower in the IF group. Both CR and IF significantly improved emotional well-being in patients with MS, notably reducing depression scores. This is offering additional emotional health benefits, which are crucial for the holistic management of MS [134]. Also, Wingo et al. [135] explored the effects of a time-restricted eating (TRE) protocols for adults with RRMS. Over 8 weeks, 12 participants ate within an 8 h window and fasted for the remaining 16 h. The findings suggest that TRE may help manage MS symptoms, mostly cognitive and motor functions. Positive participant feedback highlights that further trials are needed to confirm its effectiveness [135].

When it comes to QoL and diets, CR and fasting did not significantly impact fatigue or QoL, low-fat diets only improved fatigue, and modified MD significantly improved both fatigue and QoL [129,136]. In a systematic review by Lin et al. it was shown that IF is a promising dietary intervention for managing MS symptoms, as it can help with weight loss and enhance the QoL through mechanisms such as calorie restriction, metabolic changes, improved insulin sensitivity, better gut health, and inflammation reduction. However, variations in fasting protocols complicate the generalization of results, and the effects of IF on the immune system is not yet fully understood [137]. Therefore, extensive clinical trials are essential to explore the therapeutic potential and underlying mechanisms of IF and other diets in the context of MS.

### 3.4. Low-Salt Diet

It has recently been investigated that sodium consumption may be a dietary risk factor for the development and course of MS, which is supported by studies on mouse models. They show that a high-sodium diet increases EAE severity, enhances BBB permeability, and promotes increased numbers of peripherally generated and CNS-infiltrating Th17 cells [138,139,140,141]. The upregulated production of Th17 cells results in increased levels of interleukin-17 (IL-17), whereas reduced salt intake leads to the induction of the anti-inflammatory cytokine IL-10 [142,143]. Sodium chloride modulates the renin-angiotensin and studies indicated that a rise in systolic blood pressure induced by high salt intake is linked to disruptions in white matter integrity in young normotensive individuals [144,145].

Studies have not yet identified an association between dietary sodium intake and the risk of MS, including pediatric-onset MS [146,147,148,149,150,151]. A study observing patients with relapsing-remitting MS over a 2-year period demonstrated a positive association between MS exacerbation rates and sodium intake [149]. In addition, the evaluation of the association between urinary sodium concentration and MS progression and activity, encompassing a 5-year follow-up period, found that salt intake did not significantly impact the transformation from clinically isolated syndrome (CIS) to MS, nor did it influence clinical or magnetic resonance imaging (MRI) outcomes [150]. However, this should be confirmed and further investigated in larger studies.

## 4. Role of Probiotics, Prebiotics, and Postbiotics in MS

### 4.1. Probiotics

**Figure 4:**
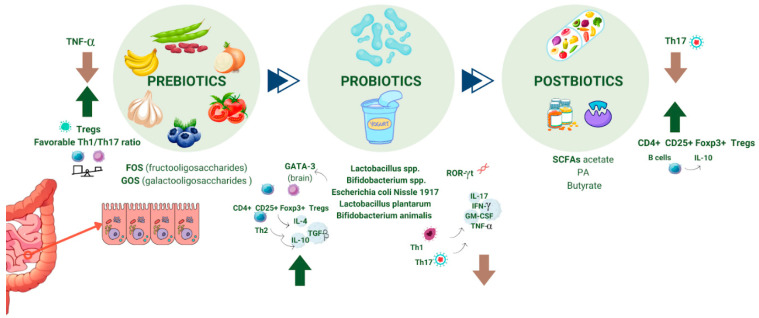
Overview of the progression from prebiotics to probiotics and finally to postbiotics, and their roles in gut health and immune modulation. Prebiotics, such as FOS and GOS, support the growth of beneficial bacteria and enhance the immune response by promoting Tregs and balancing Th1/Th17 ratios. Probiotics (e.g., Lactobacillus and *Bifidobacterium* species) further regulate immune responses, influencing Treg and Th2 pathways. Postbiotics, including SCFAs like acetate, PA, and butyrate, help maintain immune tolerance by promoting Treg activity and reducing inflammatory responses such as Th17 activation. Created with https://www.canva.com/. TNF-α, Tumor Necrosis Factor-alpha; Tregs, regulatory T cells; Th1/Th17, T helper cell subtypes; FOS, Fructooligosaccharides; GOS, Galactooligosaccharides; SCFAs, short-chain fatty acids; PA, Propionate; CD4+ CD25+ Foxp3+, markers for T regulatory cells; GATA-3, transcription factor for Th2 cells; ROR-γt, transcription factor for Th17 cells; IL-10, Interleukin 10 (anti-inflammatory cytokine).

Based on the current definition, “probiotics are live microorganisms that, when administered in adequate amounts, confer a health effect on the host” [152]. The microbiota, crucial for host survival by defending against pathogens and providing nutritional benefits, outnumbers human cells tenfold, supporting the concept of humans as “symbiotic” organisms living in harmony with their microbiota [153,154]. The ability to modulate the host’s immune responses by displacing pathogens through competitive exclusion, secreting protective mediators, and providing essential nutrients are potential benefits of probiotics, as shown in Figure 4 [153,155]. For example, before being immunized, marmoset twins were split into two groups and fed either a water-based (WBD) or yogurt-based (YBD) diet. Reduced demyelination and a weakened pro-inflammatory response from T cells, B cells, and cytokines were observed in those following the YBD diet. A few marmosets on the YBD diet showed no signs of EAE. Only after immunization did these marmosets’ gut microbiome composition change, most likely as a result of the interplay between their immune system responses and food [156].

Also, administering a combination of probiotics to mice in the chronic phase of Theiler’s encephalomyelitis virus (TMEV) infection, which is a murine model for primary progressive MS, decreased disease severity and the onset of motor disability. This treatment also influenced gut microbiota, resulting in higher levels of *Bacteroidetes*, *Actinobacteria*, *Tenericutes*, and TM7 taxa. Additionally, the study revealed reductions in gliosis, leukocyte infiltration, and the expression of IL-1β and IL-6 in the CNS, alongside elevated butyrate and acetate in plasma [157]. Also, a systematic review demonstrated that probiotics influenced the immune system by promoting the production of anti-inflammatory cytokines, such as IL-4, IL-10, and TGF-β, and regulatory T cells (Tregs), while lowering the levels of Th1 and Th17 cells and the levels of pro-inflammatory cytokines, such as IL-17, IFN-γ, GM-CSF, and TNF-α [158].

Clinical trials have also shown that administering probiotics, like capsules containing *Lactobacillus* and *Bifidobacterium* strains, resulted in improvements in disability, depression, and overall health, along with reduced expression of pro-inflammatory cytokines [89,159,160]. In their study, Tankou et al. [161] examined the effects of probiotics on gut microbiota. MS patients and healthy volunteers observed changes in microbiota composition after a two-month probiotic treatment (*Lactobacillus*, *Bifidobacterium*, and *Streptococcus*), including decreased α-diversity and increased relative abundance of the indicated species. However, stopping probiotics caused microbial and immunological alterations to reverse. The findings’ importance is limited by constraints including the small sample size, short duration, and confounding factors like dietary practices and disease-modifying medicine, which calls for more investigation [161]. *Lactobacilli also* confirmed their anti-inflammatory role by reducing Th1 and Th17 cytokines through IL-10 induction [162], as the administration of *L. casei* T2 strain led to the demyelination effects of cuprizone (CPZ) in mice [163].

### 4.2. Prebiotics

Prebiotics are products that intestinal bacteria in hosts use to support the survival and activity of particular bacterial genera and species [164,165]. They are mostly galactans and inulin [165]. Bacterial strains have the ability to break down prebiotics, which are indigestible to the host, into products that either support specific bacteria or participate in other bacterial metabolic processes, including the production of lactate, succinate, folates, indoles, secondary bile acids, and SCFA. By stimulating the growth of *Bifidobacteria* and other SCFA producers, prebiotic administration can alter the composition of the gut microbiome. Furthermore, prebiotics have the ability to activate cytokine expression and interact with immune cell receptors [166].

Therefore, prebiotics’ immunomodulatory effects rely on changes in the microbiota population through the synthesis of fermentation products like SCFAs. It is also achieved through a mechanism unrelated to the gut microbiota, as was noted by modifying B-cell responses by long-chain b2-1 fructans in germ-free mice [167,168]. Anthropometric markers, impairment levels, and measures of systemic inflammation were all correlated with dietary fiber consumption in MS patients, according to a clinical trial [169]. In addition, by altering the gut microbiota, recent research on pomegranate peel extract as a prebiotic has demonstrated that it can reduce the clinical symptoms of EAE, prevent DC activation and Th17 cell differentiation, and trigger the production of immunoregulatory cytokines, thereby establishing prebiotics as promising therapeutic candidates for further research on EAE and MS [170].

### 4.3. Postbiotics

Postbiotics are bioactive substances that are produced when fibers (prebiotics) are broken down and digested by the beneficial bacteria in the gastrointestinal tract (probiotic bacteria). Postbiotics show several advantages over probiotics in terms of safety and stability. They are safer for vulnerable populations, including immunocompromised individuals and infants, as there is risk associated with live probiotics strains. Unlike probiotics, postbiotics contain no living organisms, which enhances their consistency and reliability [171,172,173]. The primary metabolites of bacterial anaerobic fermentation of indigestible polysaccharides, such as resistant starch and dietary fiber in the human colon, are postbiotics, specifically SCFAs acetate and butyrate [174].

Recently, various studies have noted a reduction in serum butyrate levels among MS patients, which correlates with a decrease in SCFA-producing bacteria [175,176]. Additionally, MS patients show elevated acetate levels, the most abundant SCFA produced by gut bacteria [177]. SCFAs may have a similar role in MS as they do in experimental autoimmune encephalomyelitis, according to animal models [178]. It is also highlighted that SCFAs play a bidirectional role in regulating autoimmune inflammation in MS [179,180,181]. SCFAs, particularly acetate, propionate, and butyrate, have the potential to influence CNS autoimmunity, possibly via BBB transporters, highlighting their relevance in MS pathogenesis [177,182,183]. Serum levels of propionate (PA) and circulating follicular Tregs are favorably correlated with butyrate and IL-10 [184], and acetate and L-17+ [185]. In contrast, acetate levels were shown to be marginally lower in patients with RRMS or CIS, and the ratios of acetate/butyrate and acetate/(propionate + butyrate) were considerably lower in MS patients when compared to healthy controls [186].

However, research on SCFA intake for EAE and MS is still in the early stages. In experimental models, butyrate has shown to prevent CNS autoimmunity by reducing demyelination and inflammation [187]. Methyl butyrate, administered post-EAE induction, alleviated clinical symptoms and improved CNS histopathology [188]. Also, PA has been studied in both mice and humans. In EAE mice, PA intake led to a higher levels of Treg cells (CD4+ CD25+ Foxp3+), improving their clinical course compared to controls, as well as decreasing demyelination [189]. Also, in obese patients with MS, PA supplementation significantly reduced Th17 cell frequencies [190]. However, further studies and larger clinical trials should confirm the effectiveness and safety of probiotics, prebiotics and postbiotics.

## 5. Gut Dysbiosis in Pediatric-Onset MS

Analyzing the microbial composition and functional profiles of patients with pediatric-onset MS in comparison to healthy controls has been the main focus of recent studies, especially due to the technology using sophisticated sequencing techniques, such as meta-genomic analysis [76,78]. For example, the upregulation of the lipopolysaccharide biosynthesis pathway has been observed in pediatric-onset MS [76,78]. This was also noted in a study investigating the gut microbiota characteristics associated with MRI lesion burden in pediatric-onset MS [79].

In addition, breastfeeding has an important role in the gut composition in children, as it could potentially be a protective factor in pediatric-onset MS. Several studies have shown that breastfeeding promotes the growth of beneficial bacteria, such as *Bifidobacteria*, in the infant gut [80]. Breastfeeding has been shown to have an impact on the gut microbial community in late infancy, even after the inclusion of solid foods in children’s diet [80]. Breastfeeding provides infants with bioactive components that influence their microbiota, exposing them to microbial communities from breast milk and the breast surface [81]. Emerging research indicates a potential entero-mammary route for microbial transfer, suggesting that maternal probiotic supplementation could potentially modulate the gut microbiota of infants [80]. However, further studies need to investigate the role of breastfeeding in pediatric-onset MS.

### The Role of Nutrition for Patients with Pediatric-Onset MS

As previously mentioned, early life environmental factors, including diet, can influence the risk of developing MA in pediatric patients, where early lifestyle modifications can have long-lasting impacts. Research using the experimental autoimmune encephalomyelitis (EAE) model has investigated the effects of diet on MS and its progression, which is influenced by gut microbiota, enzyme activity, and vascular risk factors. There are no specific dietary guidelines for MS, although some studies indicate that a healthy diet and lifestyle improve clinical parameters and QoL [54,93]. For example, a recent study on children with early-onset pediatric MS noted that high-fat diets, especially those rich in saturated fats, were linked to a higher risk of disease progression, while sugar consumption did not significantly affect relapse risk. On the contrary, a healthy childhood diet including fruits, yogurt, and legumes was associated with a decreased risk of developing MS in adulthood [191]. Another case–control study with 95 participants (44 pediatric-onset MS cases, 51 controls) from the Canadian Pediatric Demyelinating Disease Network study examined the relationship between diet, intestinal microbiota, and MS. Participants completed a food-frequency questionnaire by age 21, and 59 provided stool samples. The findings revealed that a higher MD score and increased consumption of fiber and iron were associated with a decreased likelihood of pediatric-onset MS [77].

Diet, rather than MS, explained individual variations in gut microbiota [102]. The effects of eating vegetables and saturated fat on MS were examined in a multicenter trial involving pediatric patients with RRMS or clinically isolated syndrome (CIS). According to the study, the risk of relapsing tripled for every 10% increase in saturated fat consumption. On the other hand, the risk of relapse decreased by 50% for every extra cup equivalent of veggies. Therefore, vegetable intake provided protection, whereas fat intake was linked to an increased risk of relapse in pediatric MS, with saturated fat driving this association [103].

In the pediatric study, McDonald et al. [148] investigated the effect of different sodium intakes, but detected no significant differences in higher sodium intakes or those exceeding nutrient reference values between cases and controls. While adult and pediatric MS share similarities in presentation and pathophysiology, pediatric MS exhibits distinct clinical features and disease progression patterns. In the systematic review by Zostawa et al. (2017), it has been noted that excess sodium chloride intake may potentially contribute to inflammation in autoimmune and neurodegenerative diseases, as evidenced in both experimental and clinical settings, albeit with varying outcomes. However, current evidence suggests that adopting a low-salt diet (5 g/day) could help in preventing and treating autoimmune diseases, such as MS [151]. Nevertheless, larger clinical trials should investigate dietary interventions for pediatric patients with MS.

## 6. Conclusions

An imbalance in the gut microbiota, known as gut dysbiosis, has recently been linked to the onset and progression of autoimmune diseases, such as multiple sclerosis (MS). Diet plays a crucial role in forming the gut microbiota of a host. For example, a Western-style diet, which is characterized by high intakes of saturated fats and processed foods, has been related to the progression of MS [91]. On the other hand, the Mediterranean diet with lots of fruits, vegetables, whole grains, and healthy fats, has been linked to better clinical results and a lower risk of MS. Therefore, customized dietary approaches that target the gut microbiota by modifying immune response and gut inflammation, as well as the gut–brain axis, have the potential for more efficient management of MS. Apart from probiotics and prebiotics, diets that alter the composition of the gut microbiota offer a non-invasive and possibly economical method of MS. Despite the promising therapeutic potential of microbiota-based therapies in MS, several challenges and unanswered questions remain. One major challenge is the heterogeneity of gut microbiota composition among individuals, which complicates the identification of universal microbial targets for therapeutic intervention [33]. Furthermore, the dynamic nature of the gut microbiota and its interactions with host genetics, diet, lifestyle factors, and medications necessitate personalized approaches to microbiota modulation in clinical settings [34].

This review provides a comprehensive analysis of the interactions among gut microbiota, diet (including probiotics, prebiotics, and postbiotics), and their impact on the pathogenesis and progression of MS. Previous research has established a link between gut dysbiosis and MS, indicating that an imbalanced gut microbiota can disrupt immune responses and exacerbate inflammation. Building upon this foundation, the review explores the mechanisms through which gut microbiota modulates immune functions in MS patients. Additionally, it discusses how specific dietary patterns such as Mediterranean and ketogenic diets can influence disease progression and immune regulation in MS. The potential therapeutic role of probiotics in restoring microbial equilibrium is also emphasized based on current literature. The review consolidates evidence supporting the development of personalized dietary strategies aimed at enhancing gut microbiota as a promising non-invasive approach to managing MS. Furthermore, it investigates the reciprocal relationship between the gut and brain in MS, underscoring the need for rigorous clinical trials to validate these findings.

## Figures and Tables

**Table 1:** Effects of different dietary interventions on the immune system and gut microbiota composition.

Diet	Effect	Immune System	Gut Microbiota	References
Mediterranean diet	↑	PD-L1+ monocytes Gut Treg suppression Th2 cells	- *Lachnospiraceae* - *Bacteroidaceae* - *Barnesiella* - *Sutterella* - *Oscillospira* - *Bacteroides thetaiotamicron*	[57,104,105]
	↓	IL-17+, PD-1+ T cells Th17 cells	- *Coriobacteriaceae (Collinsella)* - *Peptostreptococcaceae* - *Ruminococcus*	
Ketogenic diet	↑	Inhibition of microglia activation (EAE) Peripheral lymphocytes count (EAE) Enzymes COX1, COX2 and ALOX5	- *Bacteroides* - *Faecalibacterium Prausnitzii* - *Akkermansia*	[106,107]
Caloric restriction	↑	Corticosterone, adiponectin (EAE) Tregs number Naïve T cells BDNF	- *Lactinobacillaceae, Bacteroiddaceae and Prevotellaceae (EAE)* - *Lactobacillus johnsonii, Lactobacillus reuteri, Lactobacillus murinus, and Lactobacillus sp. ASF360* - *Faecalibacterium* - *Lachnospiracea incertae sedis* - *Blautia*	[108,109,110,111,112,113,114,115,116]
	↓	IL-6, leptin (EAE) T cells, B cells and INF–γ (EAE) Total CD4+ T cells Pro-inflammatory cytokines (EAE) Th1 and Th17 cells (EAE) TNFα, IL-1β, CXCL2 and CXCL10 (EAE) Memory T cell effector memory reductions in Th1	- *Akkermansia*	
Low-salt diet	↑	IL-10 Treg cells	n/d	[117]
	↓	Th17 cells - IL-6, IL-23		

n/d, not defined; ↑, stimulation; ↓, inhibition.

## Data Availability

Data are contained within the article.
